# A Human Intestinal Infection Caused by a Novel Non-O1/O139 *Vibrio cholerae* Genotype and Its Dissemination Along the River

**DOI:** 10.3389/fpubh.2019.00100

**Published:** 2019-04-24

**Authors:** Songzhe Fu, Jingwei Hao, Shibo Jin, Kui Wu, Yi Wang, Shigen Ye, Ying Liu, Ruijun Li

**Affiliations:** ^1^College of Marine Technology and Environment, Dalian Ocean University, Dalian, China; ^2^College of Fisheries and Life Science, Dalian Ocean University, Dalian, China; ^3^Nanchang Center for Disease Control and Prevention, Nanchang, China

**Keywords:** *Vibrio cholerae*, genomic surveillance, movement of water, mollusc, core genomic typing

## Abstract

Non-O1/O139 *Vibrio cholerae* is increasingly reported in the clinical settings. However, intestinal infections via the consumption of non-O1/O139 *V. cholerae*-carrying seafood are rarely documented in China. In this study, we reported a case of mild watery diarrhea in a young male, caused by non-O1/O139 *V. cholerae* in the downstream of Liaohe River. Epidemiological investigation showed that this intestinal infection potentially associated with the raw consumption of mollusc. Prior to this finding, we conducted a 6-month pathogen surveillance of three locations along the Liaohe River and identified three environmental non-O1/O139 *V. cholerae* strains. To confirm the epidemiological links between clinical and environmental strains, high-resolution genomic typing was employed and revealed that *V. cholerae* isolated from human stool sample was genomically related to the one found in local mollusc and shared a common ancestor with other environmental strains obtained in the upstream sites of the Liaohe River. This fact suggests that the river is a natural reservoir for non-O1/O139 *V. cholerae* which poses a potential threat to the public health. In summary, our results deepened the insights on the transmission of non-pandemic *V. cholerae* strains and underscored the significance of genomic surveillance for drinking water along the river sites.

## Introduction

*Vibrio cholerae* is a Gram-negative, comma-shaped bacterium and belongs to genus *Vibrio*. It is a life-threatening human pathogen associated with watery diarrheal and extra-intestinal infections (1). It has been estimated that around 1.3–4 million people were infected by *V. cholerae*, resulted in estimated 21,000–147,000 deaths annually (2).

*V. cholerae* has hundreds of serogroups, but only strains belonging to serogroups O1 and O139 are recognized as the causes of pandemic transmission (3). Until now, seven cholera pandemics have been recorded since 1817 (3). The 7th pandemic erupted in Indonesia in 1961. It spread to Asia, Africa, and America by at least three independent waves of transmission and still circulated around the world (4). Recent genomic analysis indicated that cholera outbreaks between 1960 and 1990 in China were associated with wave 1 whereas later ones were mainly associated with wave 2 (5).

The non-O1, non-O139 serogroups of *V. cholerae* are only associated with a milder form of gastroenteritis, septicemia and other extra-intestinal infections. Despite of non-O1/O139 *V. cholerae* have been found in a variety of environmental sources such as wells and rivers (6, 7), the presence as well as the transmission of non-O1/O139 *V. cholerae* have not raised the concerns of the scientific community. However, in recent years, there has been increasing case reports caused by non-O1/O139 *V. cholerae* infections (8). In the US alone, around 40 cases of non-O1/O139 *V. cholerae* were reported annually to the US Centers for Disease Control and Prevention (CDC) since 2000 (2). The cases were associated with ear and wound infections as well as self-limiting and mild gastroenteritis (9). In the last two decades, at least four large epidemic diarrhea outbreaks associated with non-O1/O139 *V. cholerae* have been described. These include an epidemic report among Khmers in a camp in Thailand (10), an outbreak caused by *V. cholerae* O10 and O12 in Lima, Peru in 1994 (11), clinical cases caused by *V. cholerae* O10 in East Delhi, India (12) and a large Cholera-like diarrhea occurred in Kolkata, India (13). As current enteric disease surveillance system in China does not include non-O1/O139 *V. cholerae*, intestinal infections via raw consumption of non-O1/O139 *V. cholerae*-carrying seafood are rarely documented.

Meanwhile, the molecular epidemiology of non-O1/O139 *V. cholerae* strains remains poorly understood which severely impair our understanding of the transmission and origin of non-O1/O139 *V. cholerae* associated infection. Recently, identifying the origin of the pathogen by the use of whole-genome sequencing (WGS) is becoming a golden standard for sources tracking (14), which might help us to efficiently confirm the sources of non-O1/O139 *V. cholerae*.

Yingkou locates on delta region of the Liaohe River (Figure S1) and is an important port in North China. However, as Yingkou port has frequent trade with other regions, it was the sink of *V. cholerae* historically. In 1946 alone, there were 11 cholera outbreaks with more than 2,600 deaths (5). Recently, one clinical *V. cholerae* strain was unexpectedly identified from a shrimp farm worker in Yingkou. It remains largely unknown regarding the origins of the pathogen and its public health significance. This study aims to report this clinical case and to confirm whether the clinical *V. cholerae* strain was associated with the raw consumption of mollusc by the use of WGS.

## Case Report

A 25-year-old man who worked in a shrimp farm in Yingkou (Liaoning province, China) presented mild watery diarrhea on Aug-24, 2018. The patient presented with a previous history of raw consumption of mollusc, which was taken from the sediment in Yingkou. After 8 h of the consumption of mollusc, the patient presented with a single episode of watery diarrhea, dizziness, and vomiting. No immune-compromising disease or alcohol abuse was reported.

Over the following 2 days, he complained of abdominal pain and was treated with ciprofloxacin (500 mg every 12 h) orally. The symptom of watery diarrhea was disappeared after 3 days of antibiotic treatment. Biochemical tests revealed a high white blood cell count (14 × 10^9^/L). Stool culture was spread onto Thiosulfate-citrate-bile salts-sucrose (TCBS) and MacConkey agar plates, which gave suspected positive results for *V. cholerae*. The pure culture was obtained by a serial of sub-culture on TCBS. The suspected bacterium, namely YK-VC11 was identified as *V. cholerae* by means of VITEK-2 bacterial identifier system (BioMerieux, France) and the sequencing of the amplicon of the 16S rDNA genes (15). Agglutination with O1 and O139 antisera showed that this strain was a non-O1/O139 *V. cholerae*.

PCR was conducted for the major virulence factors of *V. cholerae* including *ompU* (outer membrane protein); *zot* (zonula occludens toxin); *tcpI* and *tcpA* (TCP expression); *hlyA* (El Tor-like haemolysin); *hapA* (haemagglutinin/protease), *rtxA* (repeat in toxin) and *toxR* (central regulatory protein) as described by Ceccarelli et al. (16). Results showed that this strain was only positive for the *toxR* and *rtxA* gene.

Afterward, we conducted WGS for this clinical strain. Genomic DNA was extracted from overnight cultures grown on TSA and fragmented and tagged for multiplexing with Nextera XT DNA Sample Preparation Kits (Illumina). The tagged DNAs were sequenced using the Illumina HiSeq 2500 at Beijing Novogene Bioinformatics Technology Co, Ltd. The raw sequencing data were submitted to GenBank (NCBI) under the BioProject No. PRJNA496566. Contigs were *de novo* assembled using SPAdes version 3.0.8 (17).

Piror to this finding we conducted a 6-month microbiological investigation of three sampling sites along the Liaohe River and identified 102 pathogenic strains from January to June 2018 (Figure S2), including two *V. mimicus* and *three V. cholerae* strains (Table 1). The identification of clinical *V. cholerae* strain promoted us to check whether this clinical case was epidemiologically associated with other environmental stains. To this end, we further selected one *V. mimicus* strain VM70 and *three V. cholerae* strains YK-VC7, HC-VC50, and AS-VC37 for WGS to confirm their epidemiological links.

**Table 1:** General features of genomes sequenced in this study.

**Species**	**Strain no**.	**Source**	**Location**	**Isolation date**	**Accession**
*V. cholerae*	YK-VC11	Human stool	Yingkou	08/2018	SAMN10491921
*V. cholerae*	YK-VC7	Mollusc	Yingkou	05/2018	SAMN10491922
*V. cholerae*	HC-VC50	River	Haicheng	06/2018	SAMN10491923
*V. cholerae*	AS-VC37	River	Anshan	06/2018	SAMN10491924
*V. mimicus*	VM70	River	Yingkou	06/2018	SAMN10491986

We first applied MLST to have a bird view of the evolutionary relationship of isolated *V. cholerae* strains. *In silico* MLST typing of 320 *V. cholerae* (Table S1) was performed by MLST 1.8 server from the Center for Genomic Epidemiology (https://cge.cbs.dtu.dk//services/MLST/) (18). Results showed that YK-VC11, YK-VC7, HC-VC50, and AS-VC37 all belonged to a new ST that is not assigned. This ST phylogenetically clustered with another unknown ST strain BJG-01, which was identified in USA (Figure S3). However, VM70 and strain 532-80 both belonged to outgroups which were distantly related to other *V. cholerae* strains.

To delineate whether four sequenced *V. cholerae* strains belonged to the same clone, first of all, 270 genomes of non-O1/O139 *V. cholerae* retrieved from GenBank (excluded strain 532-80 located in outgroup and ST69 strains) together with four sequenced strains were used for the identification of the core genome content by a method described in our previously work (19). The assembled genomes were aligned to the reference *V. cholerae* strain N16961 using progressiveMauve version 2.3.1 (20). Mobile genes, repetitive elements as well as intergenic region between core genes were excluded from the core genome. Results showed that the non-O1/O139 *V. cholerae* core genome consists of 2,683 genes (Table S2), with 2,147 genes on chromosome I and 616 genes on chromosome II.

Afterward, we combined all of the publicly available non-O1/O139 *V. cholerae* genomes with four sequenced strains and performed a phylogenomic analysis based on the non-O1/O139 *V. cholerae* core-genome SNPs. A stringent SNP calling was performed by a custom pipeline as described previously to guarantee that only genuine SNPs were included in the analysis (21). The Maximum likelihood phylogenetic trees were inferred using RAxML 7.2.8 (22). The phylogenetic relationship of closely related strains was constructed using the Maximum Parsimony algorithms in PAUP 4.0 (23).

**Figure 1:**
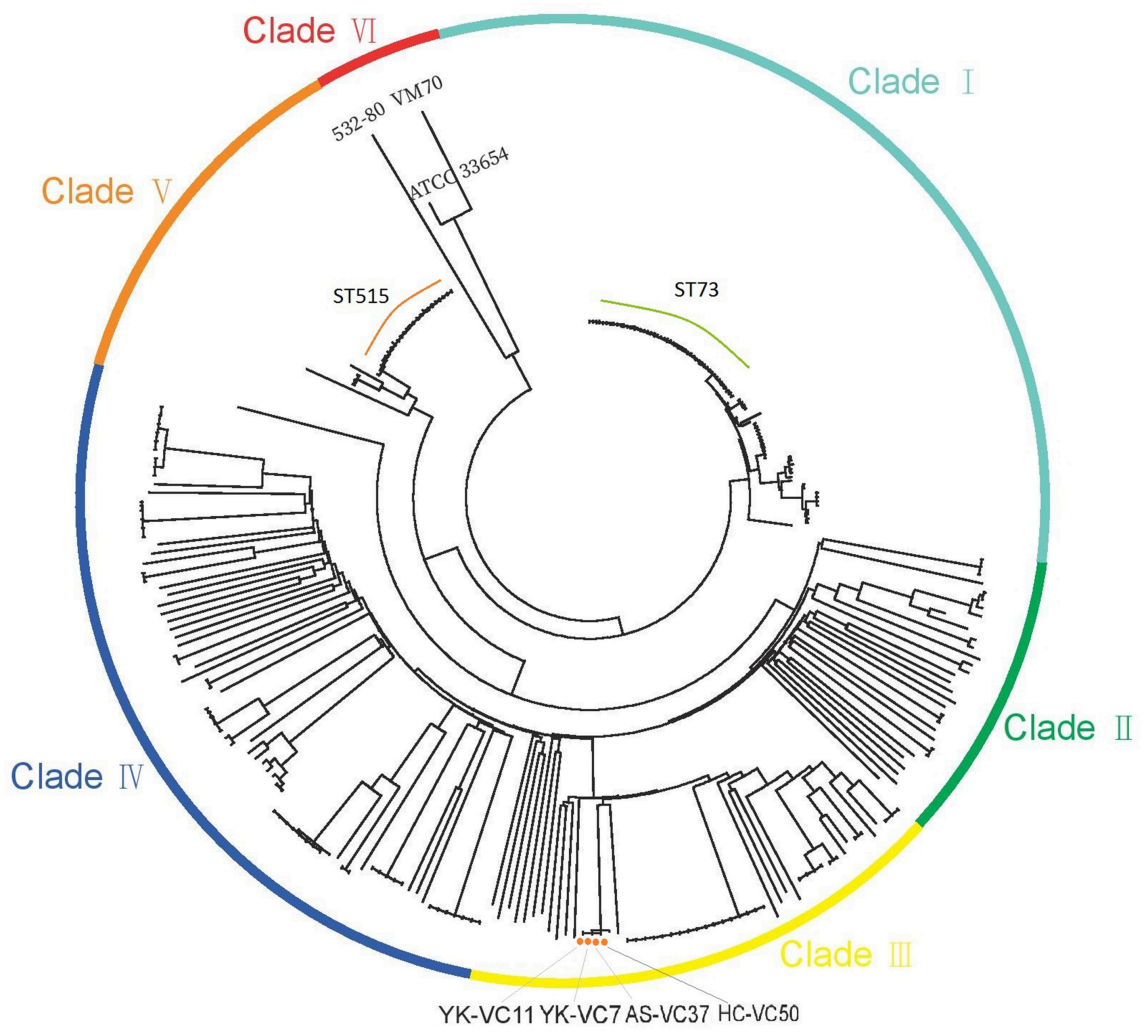
Maximum-likelihood phylogenies based on core-genome SNPs of 274 *V. cholerae* and three *V. mimicus* genomes. Colors have been grouped to reflect clade associations—Clade I (light green), Clade II (green), Clade III (yellow), Clade IV (blue), Clade V (orange), and Clade VI (red).

The SNPs alignment of analyzed genomes consists of 149,253 SNPs. The phylogenetic analysis of 277 genomes of *V. cholerae* and *V. mimicus* divided them into six clades (Figure 1). Four *V. cholerae* strains were all located within Clade III which clustered with strain BJG-01, while three genomes formed Clade VI. Notably, strain 532-80 was identified as *V. cholerae* in Genbank, but was clustered with *V. mimicus* strain VM70 and the reference *V. mimicus* genome ATCC33654. This should be corrected. Interestingly, many STs, such as ST73 and ST515 each formed a clonal cluster in Clade I and Clade VI, respectively. Strains belonged to ST73 were identified in Indonesia, Bangladesh, India, Vietnam and Kenya, while ST515 strains were circulated around Tanzania and Uganda, suggesting that some of non-O1/O139 *V. cholerae* lineages spread over the countries.

**Figure 2:**
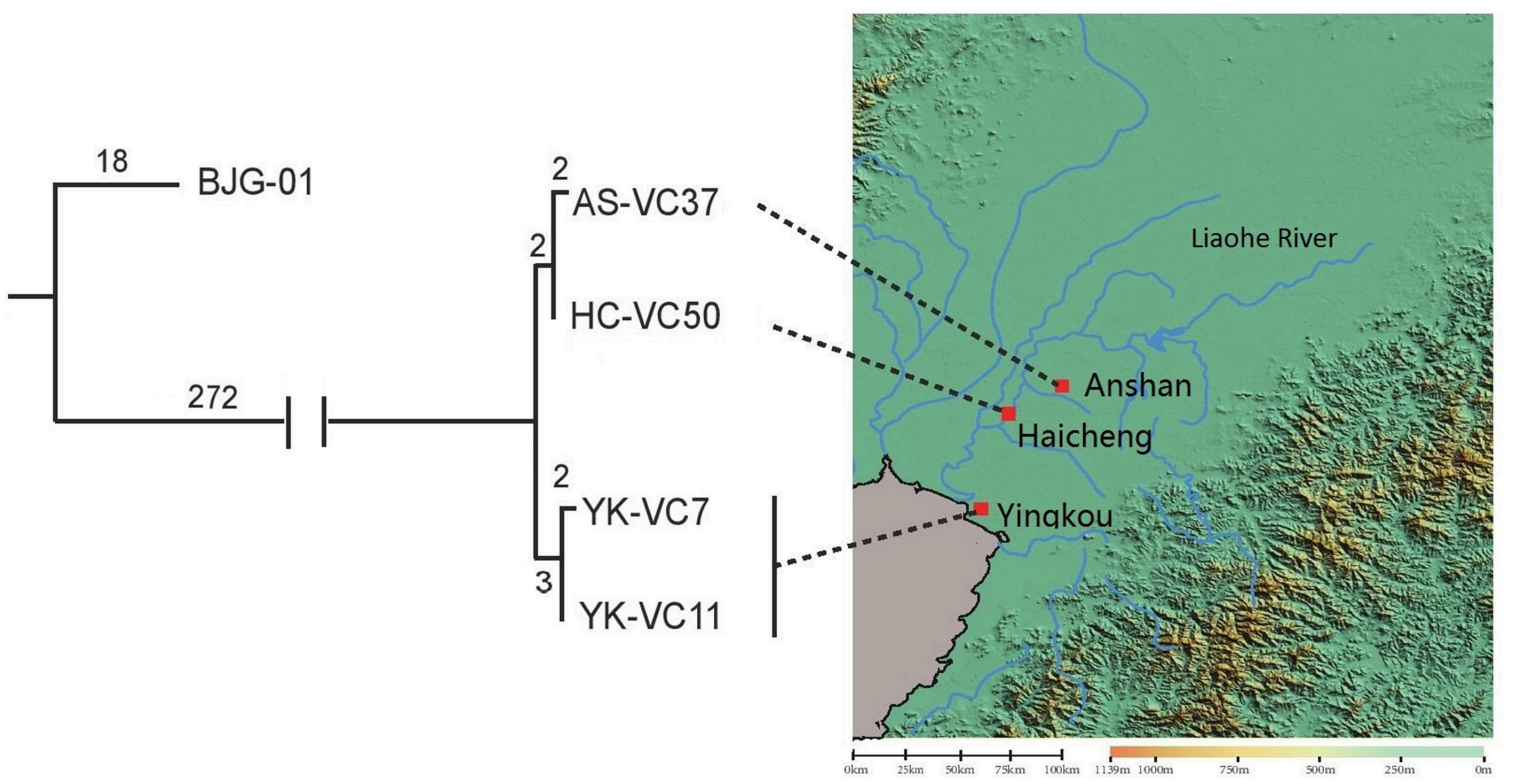
Geophylogeny of four sequenced *V. cholerae* strains. The numbers above the branches indicate the numbers of SNPs. Locations of the strains obtained from Anshan, Haicheng and Yingkou are indicated on the right side. The satellite imagery was generated by Global Mapper v20.

SNP analysis of four sequenced *V. cholerae* strains revealed that they were genomically related within 9 SNP differences at most, which represented an independent branch, differing strain BJG-01 by 290 SNPs (Figure 2). Strain AS-VC37 was clustered with HC-VC50 having 2 strain-specific SNPs, while YK-VC7 and clinical strain YK-VC11 formed another cluster with 2 SNP differences, indicating all sequenced *V. cholerae* strains belonged to the same clone.

Genomic analysis showed that all *V. cholerae* isolates found in this study were positive for *toxR* gene, but negative for *zot, ctxA, hlyA, tcpA, tcpI*, and *ompU* genes. Antibiotic susceptibility test was performed on Muller-Hinton agar (BD, USA) by disk diffusion method. Breakpoints of the antibiotics were defined according to Clinical and Laboratory Standards Institute guidelines for Enterobacteriaceae (24) and *V. cholerae* (25). *Escherichia coli* reference strain ATCC 25922 was used as a quality control strain. All strains were tested for resistance to ampicillin (10 μg), cephalexin (10 μg), cefradine (10 μg), ciprofloxacin (5 μg), chloramphenicol (30 μg), erythromycin (15 μg), florfenicol (30 μg), kanamycin (30 μg), neomycin (30 μg), penicillin (10 μg), rifampicin (30 μg), streptomycin (10 μg), sulfamethoxazole-trimethoprim (SXT) (23.75 and 1.25 μg, respectively), and tetracycline (30 μg). All of *V. cholerae* isolates were resistant to penicillin, cephalexin, cefradine, and ampicillin. However, *V. mimicus* strain conferred additional resistance to enrofloxacin, florfenicol, kanamycin, and tetracycline (Table S3).

## Discussion

In this study, we presented a case report of an intestinal non-O1/O139 infection associated with the consumption of raw mollusc. There have been numerous case reports of severe wound infections, otti media, bacteremia, and sepsis caused by non-O1/O139 *V. cholerae* (9, 26), but vibriosis case associated with syndromes of gastroenteritis is rarely documented in China. However, recent studies found that non-O1/O139 strains also caused large-scale cholera-like diarrhea in other countries, indicating it still has epidemic potential (13), which underscored the necessity for the employment of molecular epidemiology tools to delineate the origins of the outbreaks.

The genomic epidemiology of non-O1/O139 isolates has been neglected by the scientific community for many years, even if sporadic and localized outbreaks occurred each year. Octavia et al. (27) found that the majority of non-O1/O139 isolates were not genetically related by MLST. A recent analysis of 70 isolates from China also revealed that majority of STs were unique and unrelated to each other (28), which raised speculation that these lineages are likely to be the evolutionary dead ends and are unlikely to evolve into a newly emerged pathogen. Thus, they might not pose a public health threat outside the region. However, genomic analysis of publically available non-O1/O139 genomes revealed that many STs (such as ST515 and ST73) were found in multiple countries, indicating that non-O1/O139 isolates still have the potential for long-distance transmission. A major strength of our analysis is that it provides an extraordinary example to demonstrate that raw consumption of mollusc directly results in the human infection of *V. cholerae*. Raw mollusc or oyster consumption is often associated with pathogenic *Vibrio* sp. outbreaks (29). By the use of high-resolution genomic typing, our study delineated the precise origin of *V. cholerae* in human, suggesting the safety of raw mollusc or oyster consumption need to be re-evaluated.

From a public health standpoint, the findings also provided solid support for the hypothesis that the river and coastal water are the natural reservoirs for non-O1/O139 isolates which have potentials to persist in such environments. This hazard should raise awareness of the possible health risks caused by the use of river or groundwater for drinking purposes. Large cholera outbreaks in Haiti (in 2010) and many other places in the world were all due to the contamination of *V. cholerae* in the drinking water (30). As there is high-density population lived along the Liaohe River, routine monitoring should be performed for drinking water sources, which otherwise would lead to the explosive form of the outbreak. To prevent the rapid spread of *V. cholerae*, the public health department needs to strengthen the active surveillance of the drinking water sources and monitor the exact migration route of *V. cholerae*.

## Conclusion

The results herein suggest that consumption of raw or undercooked seafood is a significant transmission route of non-O1/O139 *V. cholerae* infections. The river is a natural reservoir of non-O1/O139 *V. cholerae* and may be the missing link in understanding the dissemination of non-O1/O139 *V. cholerae* within the region. This study underscored the significance of genomic surveillance along the river sites. Effective surveillance of drinking water should be taken to monitor the dissemination *V. cholerae* as well as other human pathogens along the river, which could provide valuable information for predicting future outbreaks of pathogens.

## Ethics Statement

In accordance with the Declaration of Helsinki, the patient had given his written informed consent for the use of his personal and medical information for the publication of this study. This study was approved by the ethics committee at Dalian Ocean University (permit number: 20181001).

## Author Contributions

Conceived and designed the experiments: SF and YL. Sampling and isolation of strains were performed by JH, SJ, KW, and YW. Data analysis and the draft of the manuscript were performed by SF, RL, and SY. All authors approved the final version of the manuscript for submission.

### Conflict of Interest Statement

The authors declare that the research was conducted in the absence of any commercial or financial relationships that could be construed as a potential conflict of interest.
