# Supplementary material for: A Human Intestinal Infection Caused by a Novel Non-O1/O139 Vibrio cholerae Genotype and Its Dissemination Along the River
Source: Front Public Health. 2019 Apr 24;7:100. doi: 10.3389/fpubh.2019.00100 (PMC6491804; doi:10.3389/fpubh.2019.00100)

Supplemental material

Human intestinal infection caused by a novel non-O1/O139 *Vibrio cholerae* genotype and its dissemination along the river

Songzhe Fu[1#], Jingwei Hao[2#], Shibo Jin[2], Kui Wu[3], Yi Wang[2], ShigenYe[2], Ying Liu[1], and Ruijun Li[2*]

[#] contribute equally to this paper

1. College of Marine Technology and Environment, Dalian Ocean University, Dalian, 116023, China
2. College of Fisheries and Life Science, Dalian Ocean University, Dalian, 116023, China
3. Nanchang Center for Disease Control and Prevention, Nanchang, 330038, China

Author for correspondence: Ruijun Li

Email: liruijun@dlou.edu.cn

Sonzghe Fu

Email: fusongzhe@hotmail.com

Running title: non-O1/O139 *Vibrio cholerae* in Liaohe River

Figure S1 Sampling sites in this study. The sampling positions in Anshan, Haicheng, and Yingkou are indicated in the square.

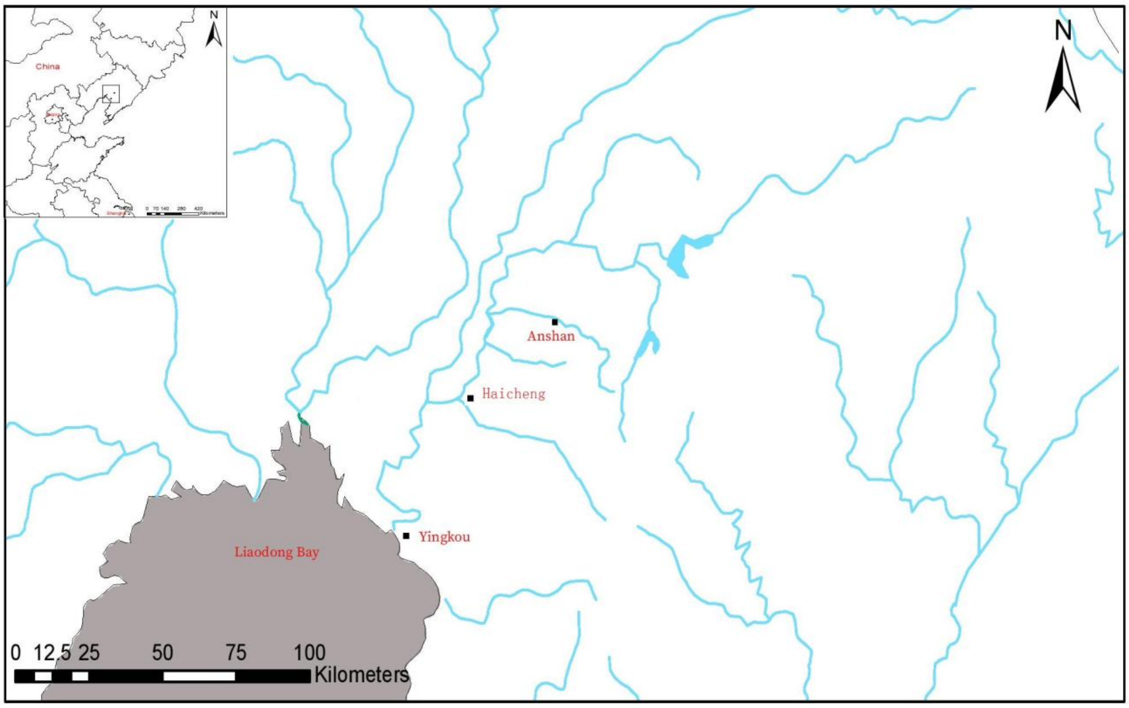

Figure S2 Pathogens isolated from water and mollusk samples in Anshan, Haicheng and Yingkou.

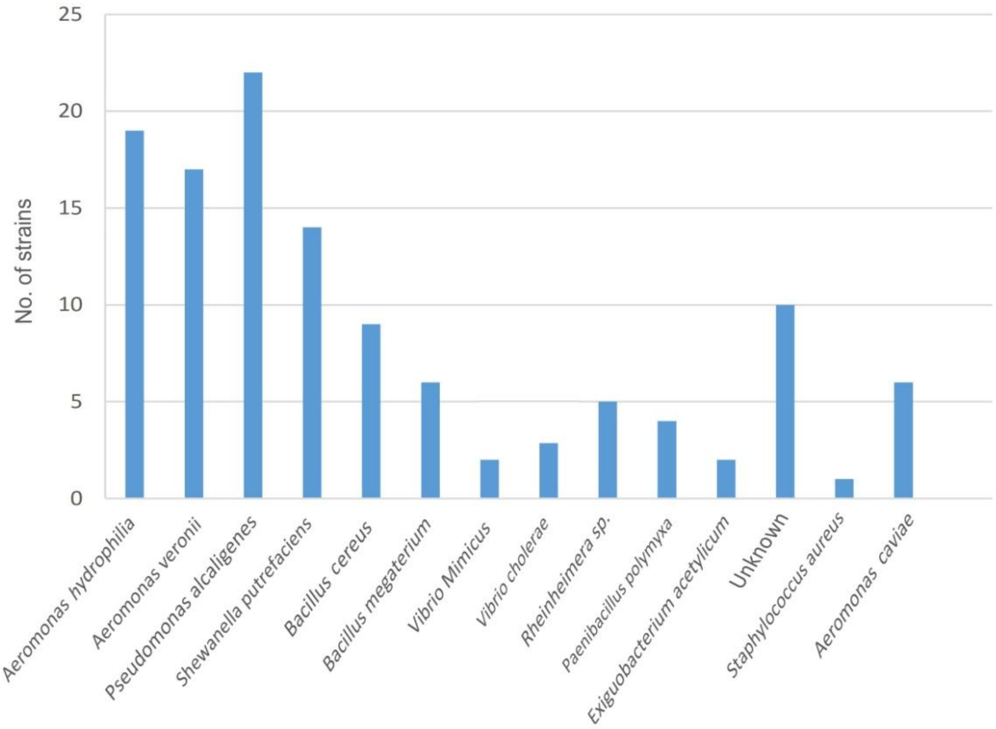

Figure S3 Neighbor-joining tree of 320 concatenated sequences of seven housekeeping genes from *V. cholerae*. The numbers at the nodes represent bootstrap values based on 1000 replications. Red square indicates the position of four sequenced strains. The phylogeny was inferred by Neibor-joining method in MEGA7.0. The genomes listed in Table S1 in the supplemental material have been included.

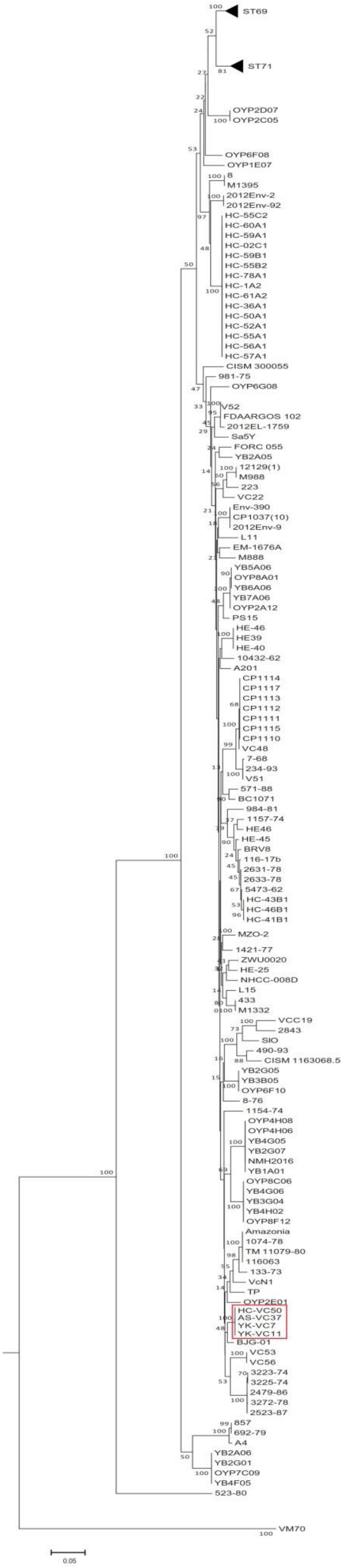

Supplement: Supplementary file 2 [file Data_Sheet_1.PDF]
